# Acknowledgement to reviewers

**DOI:** 10.1530/EC-14-A1

**Published:** 2024-12-20

The editorial board wishes to thank the following individuals who have helped to review manuscripts for *Endocrine Connections* during 2024. Their assistance is gratefully acknowledged.

Z Adamczewski

S Ahmadi

S F Ahmed

T Akamizu

K Alexandraki

M Al-Musali

F Álvarez-Nava

A S Alzahrani

N Amin

A D Anastasilakis

N M Appelman-Dijkstra

M Arumugam

A P Athanasoulia-Kaspar

M K Auer

R Auriemma

A Bachelot

C Badiu

S Bain

R Balasubranamian

I Bancos

T M Barber

M Barbot

L Bartalena

P Baumert

M Bengtsen

A Benrick

A E Berezin

M J Betz

B Bhusan

S Biswas

C Bizzarri

C S Blesson

F Bogazzi

J Bollerslev

M Bonomi

A Boot

C S Borges

M Borowczyk

H Brunetta

M Buchfelder

F Buffulo

P M Bunch

X Cai

J Calissendorff

A E Calogero

I Campi

M Cappa

L R Carvalho

E Castellano

F Ceccato

M Cerbone

H-Y Chang

I-C Chang

P Chanson

C Chatzakis

T D Cheetham

L Chen

J-H Choi

Y-M Choi

V Chortis

H L Claahsen-van der Grinten

S Clement

A Colao

G Constantinescu

M S Cooper

N Crisosto

D Cui

A F Daly

A Dastamani

M Dattani

A Dauber

S De Leo

N de Silva

N L de Silva

M Debono

J Deinum

H Deshmukh

G Di Dalmazi

D Ding

C L Doig

M D C Donaldson

J Du

I Dubinski

R Dumbell

G Effraimidis

L S Eldeiry

C Farquharson

V Favero

E T Fenta

C Finlayson

D Fintini

P Fiorina

S E Flanagan

P A Foster

T Fukuda

F Gabalec

G Gabb

S Gaberscek

E Gan

L S Gasbjerg

V A Gault

A Gawlik

G Gazdagh

N Georgopoulos

L Giovanelli

H Gleeson

V Gohil

N L R A Gomes

C H Gravholt

S Groß

H Guan

D Guensch

J Guerra

G Guney

M Gupta

O Guzman Quevedo

A Hadi

E Hammer

S-J Han

R Hardy

V Hasenmajer

E Hatipoglu

K Hawton

J J Holst

G Homuth

C M Hong

K Horiuchi

S Howard

C Hoybye

J X Hu

Z Huang

R Indirli

A M Isidori

B Jarzab

C N Jayasena

M J Jeon

M Jin

S Jin

G Johannsson

T Johnson

J D Johnston

H Jones

A P Jorgensen

C-C Juan

C C Juhlin

T Jukic

F Kalestimur

U Kampmann Opstrup

N Karavitaki

M Karownik-Lewinska

B H Kim

M Kim

S Knorr

B Kocazeybek

Z Kolesinska

P Konstantakou

S Kotschi

A Kotwal

N Kumar

C-C Kuo

C-S Kuo

F-C Kuo

A Kuś

H Kwon

C H Lee

J Lee

S H Lee

M Levy

C Li

G Li

W Li

Y Li

Y Lin

H Liu

L Liu

X Liu

Y-P Liu

Z Liu

C-H Lu

A Lucas-Herald

A Luger

D Lui

J Lund

X Luo

Y Macotela

M Madeira

P Majety

Y Mamoojee

R Mancilla

M Mao

Y M Mao

V Marotta

N Matikainen

G M F S Mazeto

G M Meduri

E Melson

E Memi

A Mena Gomez

L P Menon

L V Mihaylova

E Mills

M Mohammadnezhad

A Mondin

R Moon

L Morin-Papunen

S Mu

J Mueller

H L Müller

A Naciu

F Napoli

M Naruse

S J C M M Neggers

R Negro

R Nerenz

A Nordenstrom

T Novoselova

H F Nowotny

J Nyman

M Oczko-Wojciechowska

Y Okubo

C Ooi

M Opalińska

O Orisakwe

H-Y Ou

A S Ozgu-Erdinc

A Pal

A Panunzio

R D Paparodis

T Pappa

S A Paschou

B Passaia

Z Pei

G Pepe

R Pishdad

R Pivonello

P Popova

T Potluri

Y Pu

S Puglisi

P Puri

G Radetti

S Radmila

O Ragnarsson

M Ragonese

A Raimann

S Rao

M Read

Y Ren

R Ribeiro

S Riedl

N Rittig

E Robenshtok

V Rochira

C Romanowski

C Romei

S Roy

L Ruck

M Ruebel

J Rungby

R L Rushworth

D Rusinek

N Russell

C Sabbadin

A Sada

M Salerno

D Santovito

D Sassoon

N Sawicka-Gutaj

C Scaroni

K Schilbach

L Schock

A Sedlack

F Sesti

F Seyfried

Z Sha

G M Shaikh

M G Shaikh

F Sheikh

J Shen

J P Shield

S-R Shih

G Simeakis

F A Simmen

A Skalniak

E Søndergaard

A Sowa-Staszczak

F Spada

A Spyroglou

M Stasiak

C Stefanaki

M Stegenga

C Sturgeon

O Sverdlov

A Tabarin

M Taglialatela

K C B Tan

A Tanacan

H Tang

X Teng

A Tessnow

S Tigas

W-H Ting

G Tornese

P Touraine

N Tseng

C Tsentidis

E Tsourdi

Y-C Tung

G A Ueland

F Vaisman

E Valassi

E van Boxel

A J Van der Lely

E van Velsen

E Varaldo

V N Varlas

D A Vassiliadi

T Vatanen

M C Velarde

C Verroken

G Vila

J Wadsley

S M Wajner

Y Wang

R Wang-Sattler

M Wannack

A Weihs

D A Weinstein

S Wiegand

J M Wit

A Worthmann

T Wu

X Wu

M Xing

Y Xing

J Xu

L Xu

S Xu

W Xu

Y-S Yang

M Yavropoulou

J S Yoon

K Yu

L C Yu

M Zaidi

D E Zantut-Wittmann

G Zavatta

H-q Zhang

M Zhang

Y Zhang

H Zhao

Y Zhao

P Zheng

J Zhou

L Zhou

A Zygmunt

